# Markerless motion tracking to quantify behavioral changes during robot-assisted gait training: A validation study

**DOI:** 10.3389/frobt.2023.1155542

**Published:** 2023-03-06

**Authors:** Florian van Dellen, Nikolas Hesse, Rob Labruyère

**Affiliations:** ^1^ Sensory-Motor Systems Lab, Department of Health Science and Technology, ETH Zurich, Zurich, Switzerland; ^2^ Research Department, Swiss Children’s Rehab, University Children’s Hospital Zurich, Zurich, Switzerland; ^3^ Children's Research Center, University Children's Hospital Zurich, University of Zurich, Zurich, Switzerland

**Keywords:** lokomat, kinematics, physiotherapy, 3D gait analysis, azure kinect

## Abstract

**Introduction:** Measuring kinematic behavior during robot-assisted gait therapy requires either laborious set up of a marker-based motion capture system or relies on the internal sensors of devices that may not cover all relevant degrees of freedom. This presents a major barrier for the adoption of kinematic measurements in the normal clinical schedule. However, to advance the field of robot-assisted therapy many insights could be gained from evaluating patient behavior during regular therapies.

**Methods:** For this reason, we recently developed and validated a method for extracting kinematics from recordings of a low-cost RGB-D sensor, which relies on a virtual 3D body model to estimate the patient’s body shape and pose in each frame. The present study aimed to evaluate the robustness of the method to the presence of a lower limb exoskeleton. 10 healthy children without gait impairment walked on a treadmill with and without wearing the exoskeleton to evaluate the estimated body shape, and 8 custom stickers were placed on the body to evaluate the accuracy of estimated poses.

**Results & Conclusion:** We found that the shape is generally robust to wearing the exoskeleton, and systematic pose tracking errors were around 5 mm. Therefore, the method can be a valuable measurement tool for the clinical evaluation, e.g., to measure compensatory movements of the trunk.

## 1 Introduction

An appropriate movement control is crucial for many activities of daily living including gait. Brain dysfunctions, e.g., caused by stroke, traumatic brain injury, Parkinson’s disease, multiple sclerosis or cerebral palsy, can lead to a disturbance of gait control not only in adults, but also in children and adolescents. Typically, this means an increased risk of injuries and a restricted mobility for the affected persons. Rehabilitative interventions are often designed to practice specific movement patterns to (re-)learn movement skills, e.g., in robot-assisted gait therapy. Devices for robot-assisted gait therapy, e.g., Lokomat (Colombo et al., 2001), Gait Trainer GT (Hesse et al., 2000) or Ekso GT (Kressler et al., 2014), provide haptic guidance/resistance and enable a high number of active repetitions while reducing the physical effort for the therapist. During these interventions, therapists can formulate specific instructions, e.g., “Try to do long steps!,” to steer the patient behavior towards a more physiological gait pattern. It has been shown that persons with neuromotor impairments can react to instructions and adapt their gait pattern (Barrios et al., 2010; van Gelder et al., 2017). However, these adaptions in one joint often happen at the cost of an increased deviation from a physiological gait pattern in other joints, especially around trunk and pelvis (van Gelder et al., 2017). An understanding of these compensatory strategies could provide valuable feedback to therapists on the effects of their therapy decisions. However, this requires to measure the patients’ task performance, which is commonly related to kinematic behavior, with a clinically feasible set-up.

There are several options available to measure kinematics. In the case of robot assisted-gait training, kinematics can be often retrieved from inbuilt sensors of the device (Collantes et al., 2012). However, this approach is very limited: i) The kinematics measured by the device may not represent the actual behavior of the patient either because not all hardware adjustments are taken into account, e.g., different cuffs or sliding bars, and/or because the link between the device and the person is not fully rigid. ii) Only the angles of the sensorized joints can be estimated, but no other body parts that are also relevant for walking, e.g., trunk (Heyrman et al., 2014). In the past, gold standard methods for motion analysis based on reflective markers have been used (Hidler et al., 2008), but they also have some drawbacks. Marker-based systems are impractical as several markers have to be accurately placed on bony landmarks on the body. Due to time constraints, this is hardly feasible in a normal therapy session. In addition, the presence of the structural parts of the robot can easily occlude markers and deflect infrared signals, which leads to gaps and noisy data. Finally, marker-based systems often require multiple cameras and are too pricy for a routine use in a clinical set-up. An alternative to marker-based systems could be the use of inertial measurement units (IMUs). However, they still require the placement of several sensors. The commercially available full body tracking system Xsens (Movella, Nevada, US) for example, uses 17 sensors and has difficulties in tracking abduction/adduction (Zhang et al., 2013). Software that solely relies on 2D images can be a low-cost alternative and have already been used (Aurich-Schuler et al., 2019). Although this might be interesting for some applications, 2D methods can often only evaluate movements in a single plane of motion and are thus limited.

Methods based on RGB-Depth data have the potential to solve some of these issues. They can be based on a single sensor that is relatively cheap, e.g., Azure Kinect, and provide 3D information on the scene. From this 3D information, kinematic data can be extracted (Azure Kinect DK, 2022). Such markerless motion tracking methods are less obtrusive and can be applied during a normal therapy session with minimal setup time (Seo et al., 2019). However, the proprietary motion tracking of the Azure Kinect (K4ABT) has been developed for use cases with space and less equipment around the person of interest. Therefore, the method is strongly influenced by the parts of the robot and not useful in this context. Our own tests revealed that K4ABT failed to track the leg movements. We observed that the leg movements did not agree with the video, which was reflected in no correlation with custom tracked markers on the body surface (Pearson |r| < 0.01). Recently, we developed a method based on a statistical 3D body model that automatically adapts its pose and shape to match the person in the recorded 3D information. From the combination of pose and shape information, joint positions and angles can be inferred. This method has been validated in a gait lab against a Vicon motion capture system (Oxford, United Kingdom) (gold standard) and was superior to the proprietary tracking of Azure Kinect^1^. We expect this method to be robust against the robot parts in close proximity to the person of interest, as the statistical body model incorporates constraints on realistic body shapes.

The validation in the gait lab cannot be fully transferred to the case of robot assisted gait therapy due to the robot parts, and further analyses are necessary to ensure the validity of the method in such an environment. Therefore, the present study aims to evaluate how robust the estimation of i) the body shape and ii) the body pose is while wearing an exoskeleton.

## 2 Materials and methods

### 2.1 Participants

Ten able-bodied children and adolescents were recruited by convenience sampling between November 2021 and March 2022. Participants between 5 and 18 years old were included. Exclusion criteria were the presence of any factor that prevented the usage of the Lokomat as specified in the device’s handbook (Tölgyessy et al., 2021). Specifically, no person taller than 2 m and heavier than 135 kg or with thigh length shorter than 23 cm could participate. Participants were also excluded if they were unable to follow the study instructions or to communicate pain and discomfort. Written informed consent was provided by the legal guardian of each participant and by the participants themselves if they were 12 years or older. Ethical approval for the study was obtained from the Cantonal Ethics Committee Zurich (BASEC Nr. 21-D0044), and the study procedures were in accordance with the Declaration of Helsinki.

### 2.2 Study procedures

**FIGURE 1:**
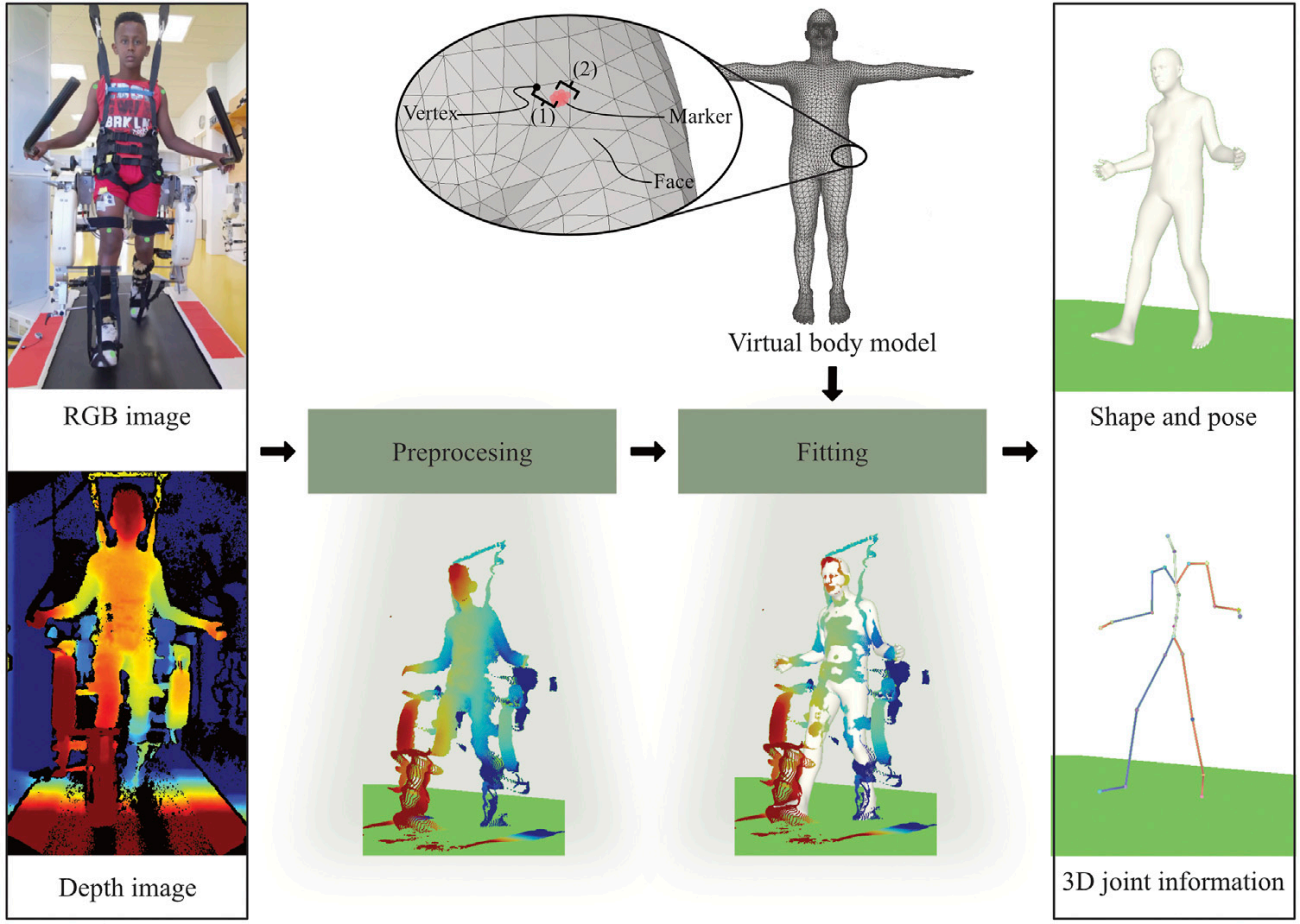
Our method takes a RGB-D recording as input and provides body shape, pose and 3D joint information as an output. The RGB image recorded by the Azure Kinect is used to segment the depth image. Consequently, the segmented depth image is converted into a 3D point cloud. Shape and pose parameters of a virtual body model are optimized to match the point cloud. The virtual body model consists of triangular faces and vertices (corners). For the validation, the distance between each sticker and the nearest vertex on the mesh is calculated. As the sticker might not be placed exactly on a vertex, the total error consists of an offset (to the closest vertex) and the actual fitting error.

The participants walked for a total of 20 min in the robot-assisted gait trainer Lokomat followed by 3 min of treadmill walking. The last minute of each condition was recorded with an Azure Kinect (Microsoft, Seattle, United States) placed approximately 1.5 m in front of the participants in portrait view. The Azure Kinect includes both a RGB sensor (1920 × 1080 px, 30 fps) and a time of flight depth sensor (640 × 576 px, 30 fps for the unbinned narrow field of view mode). Two red stripes were placed on the treadmill to identify a floor plane, and bright green stickers were placed on eight prominent landmarks of the body (each left and right, acromioclavicular joint, anterior Spina iliaca, patella and hallux) as reference points for the evaluation of pose tracking accuracy (Figure 1). These landmarks were selected based on three main criteria: (a) Landmarks are easy to detect and thus the placement of markers is replicable, (b) Landmarks are close to joint centers, which could be influenced by the presence of the robot, (c) Landmarks are visible in the RGB-D images at angles, which can be reliably detected (Tölgyessy et al., 2021). Therefore, no marker could be placed directly on the ankle joint.

### 2.3 Data processing

Throughout the data processing, the sequence of RGB-D images was converted into kinematic information described by the 3D body model, i.e., body surface and joint kinematics. Data processing and fitting was performed on a XPS Desktop PC (Dell Technologies Inc., Texas United States ) with a GeForce RTX3070 graphics card (NVIDIA Corporation, California, United States).

#### 2.3.1 Preprocessing

The recordings were unpacked, and RGB images were downscaled and aligned with the depth image using the Azure Kinect SDK, and then converted into 3D point clouds (Azure Kinect DK, 2022). We considered the first 900 frames per recording, which equals to 30 s. All further steps were implemented and computed with Python 3 (Python Software Foundation, Beaverton, United States). The 3D point clouds were segmented into person of interest and background by using an estimated ground plane and simple distance thresholds. The robotic parts in close proximity of the body could not be reliably detected, and were therefore not removed.

#### 2.3.2 Statistical body model

The body surface of a statistical body model is represented as a mesh, which consists of triangular faces, which are defined by the three corners, so-called vertices (see Figure 1). Consequently, the shape and pose of the body can be changed with two sets of parameters, which have been learned from a large number of high resolution body scans. Here, the *Sparse Trained Articulated Human Body Regressor* (STAR) body model is used, which is freely available for research purposes (Osman et al., 2020), and provides 10 parameters to adapt the body shape and 72 parameters to adjust the body pose. The pose parameters correspond to three degrees of freedom for 23 body joints and one root joint. As STAR was learned from adult body scans only, it can have difficulty to cope with the different body proportions of children. This was solved by interpolating STAR with a skinned multi-infant linear model, SMIL (Hesse et al., 2020). As an additional constraint, the model height was fixed to the actual height of the participants.

#### 2.3.3 Model fitting

The statistical body model was registered to the point cloud by optimizing shape and pose parameters of a virtual body model with a method based on (Hesse et al., 2020). The source code for the model fitting will be made available to the public (https://github.com/nh236/smplify-kids). The main objective of the optimization was to minimize the distances between points of the 3D point cloud and the model surface. To improve the method for the present use case, three modifications were made. i) As the shape of the shanks and the feet was altered by the robot’s foot straps, which secure a proper foot lift during the swing phase, we temporarily added an auxiliary model of the foot straps consisting of 8 vertices and 6 degrees of freedom. ii) Shape parameters were only optimized during the first five frames, after which the shape was fixed to the average of these five initialization frames, and only the pose was optimized in the rest of the frames. iii) Two additional terms were introduced and added to the objective function. Firstly, movements of the auxiliary vertices were allowed, but controlled with a loss. Secondly, excessive flexion of the ankle was limited by a quadratic loss dependent of the angle. A detailed description of the optimization and the weights of the different loss terms can be found in the Supplementary Material.

#### 2.3.4 Sticker tracking

Due to the aforementioned issues with marker-based motion capture systems, they are difficult to be used during robot-assisted gait therapy. In addition, in a previous study we found that reflective markers distort the body surface^1^. Both issues together limit a fair comparison of the method in our case. Instead, we used colored stickers, which we detected and tracked in the 3D data as a reference for evaluating the accuracy our method. The accuracy of the measurement of the Kinect Azure was found to be reliable for the distances in the context of this study (Tölgyessy et al., 2021). The eight stickers were segmented in each RGB image using a color filter and the corresponding 3D locations of the pixels were obtained from the aligned depth image. Subsequently, the final sticker position was estimated from the median position of all the 3D points forming one sticker. This resulted in a 3D time series per sticker acting as an reference point for a body part in the 3D data. In some cases, stickers were not correctly detected (e.g., through an arm movement masking the sticker in the image). This led to missing data and the corresponding segment of the time series was not included in the analysis.

### 2.4 Statistics

As the position of the joint centers is inferred from the body shape, robustness of both shape and pose against noise and additional data points belonging to the robotic device were considered.

#### 2.4.1 Model shape comparison

**FIGURE 2:**
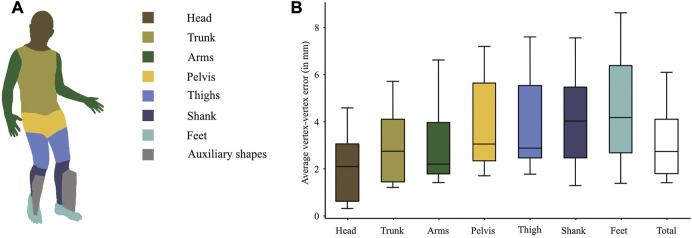
**(A)** illustrates the regions on the body model for which the average vertex-vertex distances per subject were calculated to compare the model shapes between walking with the robot and walking on a treadmill. The boxpots show medians and IQRs across subjects. The auxiliary shapes were only temporarily introduced and are thus not part of the evaluation. **(B)** The boxplots show the average distances per segment across the 10 subjects. The total shape difference is the mean across all vertices.

The model fitting is mainly based on the minimization of the distance between each data point and its closest vertex on the body model. Points in the data that do not belong to the person of interest might “pull” the model towards them and lead to an overestimated size of the person’s shape. Our aim was to evaluate the influence of robot parts in close proximity to the human on the fitting result by comparing the body shapes between the treadmill walking (without robot) and robot-assisted walking. To this end, we computed the mean distance between corresponding vertices, i.e., vertices with the same vertex ID of the two fitted models, for seven body parts (see Figure 2A). Then, the median and interquartile range per segment across all participants were computed.

#### 2.4.2 Model-sticker distance

Besides influencing the body shape, the additional points could also lead to pose errors by drawing the model towards the robot parts during fitting. To this end, we used the stickers on the body parts as reference points in the 3D point cloud, from which the model would deviate if it was drawn away from the true body position. It was not possible to predetermine one vertex for all participants, as the vertex matching the landmarks might vary due to different body types. Therefore, we determined the vertices with the smallest Euclidean distance to the corresponding reference point in each frame. A single vertex, which matched the marker best across all frames, was selected. In addition, the agreement of this selected vertex with the corresponding landmark was visually verified. The same vertex was used to evaluate the model regarding shifts with respect to the corresponding sticker for all frames. As the position of the reference points might not exactly coincide with a vertex, the evaluation of the absolute difference between the reference point and its closest vertex, e.g., by a root mean square error, includes a systematic offset (see Figure 1, total error). In case of a perfect fitting, the anchor and the closest vertex would always move together. Therefore, we evaluated the fitting error by measuring the fluctuation of the distance, i.e., the standard deviation, between each reference point and its closest vertex in combination with 3D correlation of the signals (Pearson’s r). The error was further decomposed in a random component, which can be removed by segmenting the data into strides and averaging a sufficient number of strides, and a systematic component, which occurs at a harmonic frequency of the gait cycle and can be extracted with a filter that lets these frequencies pass. A good tracking quality would therefore result in a low systematic error, in combination with a high correlation coefficient. To get an estimate of the distribution of the error magnitudes, we calculated the percentage of samples with an error below 5 mm, 10 mm, 20 mm, 30 mm 50 mm and 100 mm for each sticker individually. The results of the left and right side were averaged.

## 3 Results

None of the 10 subjects had to be excluded. The subjects covered a wide range of body heights ranging from 1.25 m to 1.76 m. The computation time for the fitting typically ranges between 1 and 2 s per frame.

### 3.1 Model shape

The shape comparison (see Figure 2) revealed a median difference between corresponding vertices of 2.7 mm between treadmill walking and Lokomat walking. Eight of the 10 subjects had an average vertex-to-vertex distance of less than 4 mm. The difference was smallest at head, trunk and arms and largest at the distal segments shanks and feet (see Figure 2B). For the feet, which showed the largest shape deviations, the shape difference was smaller than 7 mm in 8 out of 10 subjects.

### 3.2 Model-sticker differences

**FIGURE 3:**
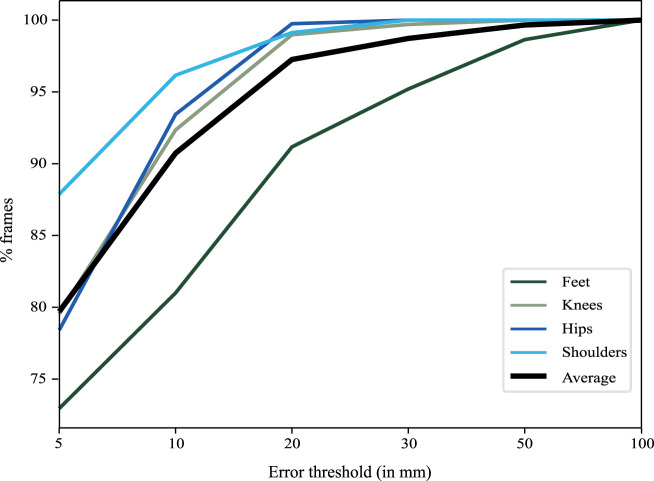
The graph illustrates the percentage of frames (*y*-axis) below a certain error threshold (*x*-axis). 95% of the frames had an error smaller than 2 cm with the errors around the feet being slightly larger than elsewhere.

The differences between the stickers and the model are smallest around the trunk (∼4 mm) and largest at the feet (∼15 mm) (Table 1). Similarly, the systematic error is largest at the feet but less than 5 mm for the rest of the body parts (Table 1). At the feet, errors larger than 50 mm occur in 1%–2% of the frames. In 80% of the frames, the errors are smaller than 10 mm (Figure 3).

**TABLE 1:** Sticker to model error, systematic error in millimeters & Pearson correlation coefficient averaged across subject.

Segment	Mean error (standard deviation)	Mean systematic error (standard deviation)	Pearson correlation coefficient
Feet	14 (6.2)	8.8 (7.9)	0.99
Knees	6.8 (1.8)	4.8 (2.2)	0.99
Hips	6.2 (1.9)	3.2 (1.6)	0.99
Shoulders	4.6 (2.5)	1.6 (1.3)	0.99
**Average**	**7.9 (5.1)**	**4.6 (4.9)**	**0.99**

## 4 Discussion

### 4.1 Model shape

The results show that most of the errors are in the range of 4 mm for differences in shape, and largest around the feet (maximum around 8 mm). This difference is small considering that at a distance of 1.5 m from the camera, 4 mm are equal to a single pixel. The difference is largest around the feet, and there are two reasons why this might be the case. Firstly, the shape parameters are determined during the first few frames and then kept fixed to avoid drifts in the body shape instead of kinematic changes. However, the initialization was not tied to a certain foot position and therefore the initialization could have happened close to heel strike in one condition (e.g., treadmill walking) and close to toe off in the other condition (e.g., robot-assisted walking). This would change the point of view of the foot and might have had some impact on the fitting result. Secondly, the foot straps of the robotic device partially occluded the view of the foot, introducing noise in the point cloud around the shanks and the feet. Although partially modeled by the auxiliary shapes, this is the biggest difference to the treadmill condition. Despite the comparably larger errors around the feet, it is unlikely that these differences of a few millimeters bias the fitting results such that they impact the interpretation of extracted kinematic patterns.

### 4.2 Model-sticker differences

The comparison of vertices to the stickers showed small tracking errors together with almost perfect correlations. Thus, the point cloud and the model move together. Only in less than 1%–2% of the frames, the feet lost the point cloud illustrated by errors larger than 5 cm. A substantial portion of the observed errors is due to noise and could be reduced by handling gait recordings with standard procedures like segmentation into gait cycles, registration and averaging of multiple strides (Chau et al., 2005). For example, 10 steps would be required to detect a difference in step length of 1 cm with a statistical significance of 0.05 and a statistical power of 0.8 for a *t*-test based on the measured variability. However, the analysis also revealed that a systematic error (with a harmonic frequency of the gait cycle) remains even if an infinite number of strides would be used. This is especially apparent at the knee and the feet. A visual inspection of the recordings revealed that this error occurs especially around toe-off. In this position, the foot is the furthest from the cameras, and, also due to the noise introduced by the foot straps, less points are available to obtain an accurate fitting of the foot. However, due to the very high correlations between the stickers and the corresponding model vertices, this does not pose a problem for comparisons measured with the same tracking method. Nevertheless, users should be aware of this issue, which mostly affects the distal body parts. The distribution of the tracking errors shows that the foot of the model loses the point cloud only in very few frames (1-2 out of 100). Considering that the typical cadence is around 1.2 Hz (Winter 1984) and the frame rate of the Azure Kinect is 30 frames per second, this occurs in one frame every three strides and the tracking can be considered robust.

Advancements in the field of computer vision might further increase the accuracy of the fitting. On the one hand by improving the models, especially around the feet [e.g., SUPR (Osman et al., 2022)], and on the other hand by improving the pose estimation (e.g., by improving the optimization described in section 2.3.3).

An advantage of the method presented here is that it is based on a shape prior learned from a large number of high-resolution depth scans (Tölgyessy et al., 2021). The model can therefore deal well with occlusions that involve only smaller parts of the body. For example, the model coped well with Bodyweight Support System straps or cuffs around the thighs. This strength is what makes the method presented here so interesting for use in the context of human-robot interfaces. However, occlusions were more problematic when they covered a significant portion of a segment in close proximity to a joint. In the present study, the Lokomat occluded a portion of the lower leg and ankle, resulting in an unstable fit. Therefore, auxiliary shapes were introduced to improve the accuracy of fitting around the ankle. These auxiliary shapes were treated as an additional body part during optimization before being removed prior to analysis. In this use case, the auxiliary shapes improved the stability of the fitting. This observation has some implications for users who want to adapt the method for other devices: (a) First and foremost, the position of the camera should be chosen to avoid occlusions as much as possible. (b) Small occlusions (e.g., cuffs in the middle of the lower or upper thigh) are not problematic because the shape prior prevents strong distortions. (c) The influence of larger occlusions and occlusions around joints can be mitigated by temporarily including a 3D model of the occluding part in the optimization. Since many exoskeletons use a similar fixation system as the Lokomat, based on cuffs and straps, we assume that the presented results are transferable to other devices (Osman et al., 2020).

### 4.3 Limitations

It is important to consider some limitations that are associated with this study. First of all, the tracking method was not validated against the gold standard in this set-up as this is associated with issues mentioned before. Instead, both reference and the method rely on RGB-D images. However, the comparison did not focus on whether the measurement of the RGB-D camera is valid, as it has already been shown that the method performs well (without robot) in comparison to a gold-standard motion tracking system^1^. Instead, our comparison focused on whether the method and the placement of the model in the point cloud was robust to the presence of an exoskeleton. On the one hand, the model used to track the movement relies on a large number of points and is therefore less sensitive to noise in the RGB-D image, but there is no clear attribution which points do or do not belong to the body. On the other hand, the tracking of the stickers relies on less points and is therefore affected more by the sensor noise, but it can be clearly defined which points do or do not belong to the stickers. Therefore, the tracking of the stickers is not affected by the robot, which allows to use them as a reference (Seo et al., 2019) We cannot rule out that some of the error can be also attributed to the tracking of the stickers. However, a recent study found that the depth noise of the Azure Kinect was around 2 mm at the distances relevant in this study (Tölgyessy et al., 2021). In addition, the robust statistics used to compute the center of the marker limit the influence of noisy data. Therefore, large deviations which would impact the interpretation can be excluded. The focus of the present study was to demonstrate that this method is robust to the presence of robot parts for gait training and the method can be used to obtain kinematic measures in a clinical set up with minimal obtrusion of the normal clinical workflow.

Furthermore, a limitation of the method is that the optimization can get stuck in a local minimum. This is dependent on the noise in the data and can be a problem when only few data points are available during the initialization. This is especially relevant if the foot is far away from the camera and covers fewer pixels, while in case of correct initialization of the model, errors rarely occur as demonstrated above. As a consequence, the initialization stage needs to be manually verified, and an automated selection of eligible initialization frames needs to be developed to make the pipeline fully automatic. However, time demands and skills required to set up the measurement system are very low and it is possible to record the kinematic behavior in a clinical environment without interfering with the therapy procedures. Thereby, the measurement can be done without a contribution from the patient and the therapist and the majority of the workload occurs during data processing, which is a major advantage over other methods and the gold standard.

Lastly, the final stage, the inference of the joint positions was not part of the analyses in the absence of a gold standard with the exoskeleton. However, this stage depends only on the results of the previous stages. Therefore, it is not influenced by the presence of an exoskeleton and was already covered elsewhere^1^.

## 5 Conclusion

We were able to demonstrate that our motion tracking method is able to record accurate data sets of human kinematics of children walking in an exoskeleton. This method has substantial advantages over marker-based motion tracking systems, as no contributions of therapists and patients are required. This is beneficial for measurements in a clinical environment and might therefore improve the acceptance by therapists and compliance by the patients. The virtual body model-based tracking enables researchers and clinicians to evaluate trunk compensatory movements, quantitative evaluation of improvements within the system or reactions to therapeutic instructions and could thereby help to improve the effectiveness of the therapies.

## Data Availability

The raw data supporting the conclusion of this article will be made available by the authors, without undue reservation.

## Footnotes

1 Hesse, N., Baumgartner, S., Gut, A., and van Hedel, H. J. A. (2023). Concurrent validity of a custom method for markerless 3D full-body motion tracking of children and young adults based on a single RGB-D camera. Trans. Neural Syst. Rehabilitation Eng. 2023.

## Abbreviations

IMU, inertial measurement unit; SMIL, skinned multi-infant linear model; STAR, sparse trained articulated human body regressor; SUPR, sparse unified part-based human representation.
