# Supplementary material for: Markerless motion tracking to quantify behavioral changes during robot-assisted gait training: A validation study
Source: Front Robot AI. 2023 Mar 6;10:1155542. doi: 10.3389/frobt.2023.1155542 (PMC10025461; doi:10.3389/frobt.2023.1155542)
Supplement: Supplementary file 1 [file Table1.DOCX]

**Supplementary Material – Tracking Pipeline**

We here present the tracking pipeline in more detail together with a table specifying the used weights to ensure the possibility to reproduce the results. For a full description, we want to refer readers interested in applying the method to the original paper as cited in the manuscript. To segment the child from the treadmill and the background, points belonging to the treadmill were identified with a color mask (red stripes placed on the treadmill) and then a floor plane was fit to them using RANSAC. All data that were equal or lower than that floor plane were removed from the point cloud. Furthermore, all data closer than 0.5m and more than 3m of the camera were excluded. All remaining points for the further steps could be either attributed to the participant or the exoskeleton. The floor plane was also used during the fitting to restrict the foot soles during stance phase to ground contact.

The fitting of the model parameters to the 3D point cloud was split into three stages that differed in the weights of the terms in the objective function and the tuned parameters of the model. During the two initialization stages, the model center was first set to the center of the point cloud and 2D keypoints from the RGB images (12) were used to initialize the body pose (E_keypoints2D_). Only during the first 5 Frames, both pose and shape parameters were optimized. For all other frames, new weights were used and the average shape parameters of these 5 initialization frames were fixed and only the pose was optimized in the rest of the frames. The objective function minimized during optimization was dominated by the sum of the squared distance of each point to its closest vertex (E_s2m_). To ensure that no model part loses the mesh an additional loss term was added consisting of the sum of the squared distances of each visible vertex to the closest point in the 3D point cloud (E_m2s_). For both terms the influence of noise (e.g. due to the exoskeleton) was reduced by only considering distances below a threshold (e_s2m,_ e_m2s_). As human movements are generally smooth deviations in pose from a previous frame to the next frame were penalized with a L2 loss (E_smooth_). Also ground contact during walking was ensured by a L2 loss term “pulling” the foot sole vertices to the floor plane if they are closer than 5cm to the floor plane (E_plane_). Self-penetration of the mesh (E_collision_) and points inside the ground plane (E_inside_) were penalized. Furthermore, the movement of the auxiliary vertices was allowed but penalized with a loss (E_auxiliary_). Excessive flexion of the ankle was limited by a L2 loss of the angle (E_footpose_). A gaussian mixed model pose prior (13) was used to avoid extremely unrealistic poses (E_pose_).

| Name | Initialization 1 | Initialization 2 | Weights |
| --- | --- | --- | --- |
| e_m2s_ | 0.3 | 0.03 | 0.03 |
| e_s2m_ | 0.3 | 0.03 | 0.03 |
| E_keypoints2D_ | 1.0 | 1.0 | 0.025 |
| E_m2s_ | 40000.0 | 40000.0 | 40000.0 |
| E_s2m_ | 100000.0 | 200000.0 | 900000.0 |
| E_collsion_ | 0 | 0 | 0.3 |
| E_smooth_ | 1.0 | 1.0 | 0.5 |
| E_plane_ | 10000.0 | 1000.0 | 6.0 |
| E_inside_ | 1000000.0 | 1000000.0 | 10000.0 |
| E_footpose_ | 1.0 | 1.0 | 10.0 |
| E_pose_ | 1.5 | 1.5 | 0.1 |
| E_auxiliary_ | 8000.0 | 8000.0 | 8.0 |
